# Divergent Effects of Cytomegalovirus and Rheumatoid Arthritis on Senescent CD4^+^ T Cells

**DOI:** 10.1002/eji.70093

**Published:** 2025-11-14

**Authors:** Lea Williams, Ali O. Saber, Silina Awad, Xi Su, Asgar Ansari, Ruozhang Xu, Hannah Jung, Anupama Shahane, Joshua F. Baker, Laura F. Su

**Affiliations:** ^1^ Department of Medicine Division of Rheumatology Perelman School of Medicine Institute for Immunology and Immune Health University of Pennsylvania Philadelphia Pennsylvania USA; ^2^ Corporal Michael J Crescenz VA Medical Center Philadelphia Pennsylvania USA

## Abstract

Chronic antigen exposure drives CD4⁺ T cell senescence, yet how autoimmunity and persistent viral infections differentially shape T cell differentiation and function remains unclear. Using cytomegalovirus (CMV) and rheumatoid arthritis (RA) as models of chronic immune activation, we performed high‐dimensional mass cytometry and functional assays to define their impact on CD4⁺ T cells. In CMV‐seropositive individuals, CD27^−^CD28^−^ CD4⁺ T cells were abundant and exhibited a predominantly cytotoxic, nonproliferative phenotype. Only a minor fraction was CMV‐reactive, suggesting that bystander‐driven differentiation contributes to this subset. In the absence of CMV, senescent CD4⁺ T cells were infrequent and phenotypically distinct, though they still exhibited low proliferative capacity. EBV and HSV did not independently increase CD27^−^CD28^−^ CD4⁺ T cell frequency. Similarly, RA had little effect on their abundance but instead tuned the functional quality of senescent cells. In CMV‐seropositive RA patients, senescent CD4⁺ T cells produced less pro‐inflammatory cytokines and showed impaired cytotoxic degranulation. Central memory CD4⁺ and CD27^−^CD28^−^ CD8⁺ T cell functions were preserved, with no evidence for CMV reactivation, suggesting maintained viral control by unaffected T cell responses. These findings highlight distinct, nonredundant effects of CMV and RA on CD4⁺ T cell senescence and reveal RA‐specific functional defects in senescent CD4⁺ T cells.

## Graphical abstract

How chronic immune stimulations differentially shape CD4⁺ T cell senescence remains unclear. Using high‐dimensional mass cytometry and functional profiling, we show that cytomegalovirus (CMV) drives expansion of cytotoxic CD27^−^CD28^−^ CD4⁺ T cells, whereas rheumatoid arthritis (RA) exerts nonredundant effects by modulating their effector capacity.

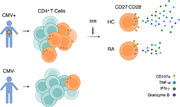

## 1 Introduction

Chronic antigen stimulation drives terminal T‐cell differentiation. In patients with rheumatoid arthritis (RA), a highly differentiated CD4^+^ T cell population has been linked to increased disease activity, joint damage, and cardiovascular disease [1, 2, 3, 4, 5]. These end‐differentiated CD4^+^ T cells, exhibiting replicative senescence, frequently lacked costimulatory receptors and expressed CD57, a terminally sulfated carbohydrate epitope [6]. They are highly proinflammatory, producing IFN‐γ and TNF‐α and expressing cytotoxic proteins, resembling cytotoxic CD8^+^ T cells [7].

Similar phenotypic subsets have been identified in other autoimmune diseases, infections, and cancer [8]. For example, a cytotoxic CD27^−^CD28^−^CD4^+^ population emerges after primary cytomegalovirus (CMV) infection and is expanded during viral latency [9, 10]. An accumulation of replication‐impaired CD57^+^CD4^+^ and CD8^+^ cells has also been observed in HIV infection [11, 12]. In contrast to their presumed pathologic roles in autoimmunity, cytotoxic CD4^+^ T cells can be protective and have been shown in mice to provide direct defense against lethal influenza infection [13]. In a human influenza challenge study, a higher baseline frequency of cytotoxic CD4^+^ T cells correlated with decreased viral shedding, lower symptom scores, and reduced disease duration after exposure [14]. Recent studies on tumor‐infiltrating T cells have also shown that cytotoxic CD4^+^ T cells play a critical role in tumor surveillance, with the ability to kill tumor cells in a class II MHC‐dependent manner [15, 16, 17, 18]. In supercentenarians, these cells are significantly more abundant than in younger old adults and are thought to enhance antitumor and antiviral immunity [19].

These studies highlight the various context‐dependent roles of this unique CD4^+^ T cell subset. However, in the complex human environment, exposures seldom occur in isolation. How multiple co‐existing stimuli converge to influence T cell baseline states remains unclear. Using high‐dimensional mass cytometry, we analyzed CD4^+^ T cells from CMV seropositive and seronegative individuals, with and without RA, to investigate disease‐ and infection‐related changes in T cell differentiation. Our data identified CMV as a key driver of senescent differentiation in CD4^+^ T cells. In the absence of CMV, CD27^−^CD28^−^CD4^+^ T cells were infrequent, irrespective of RA status or EBV and HSV infections. However, CD27^−^CD28^−^ CD4⁺ T cells in CMV‐seropositive RA patients exhibited altered function, marked by reduced cytotoxic degranulation and cytokine production. These functional impairments were largely restricted to the CD27^−^CD28^−^ CD4⁺ subset. In contrast, effector responses in other T cell subsets, including central memory CD4⁺ and terminally differentiated CD8⁺ T cells, did not differ significantly between RA patients and controls. Together, these findings highlight CMV as a major driver of CD4⁺ T cell senescence and reveal RA‐specific modulation of CD4⁺ T cell function.

## 2 Results

### 2.1 CMV Drives the Expansion of CD27^−^CD28^−^ T Cells

**FIGURE 1:**
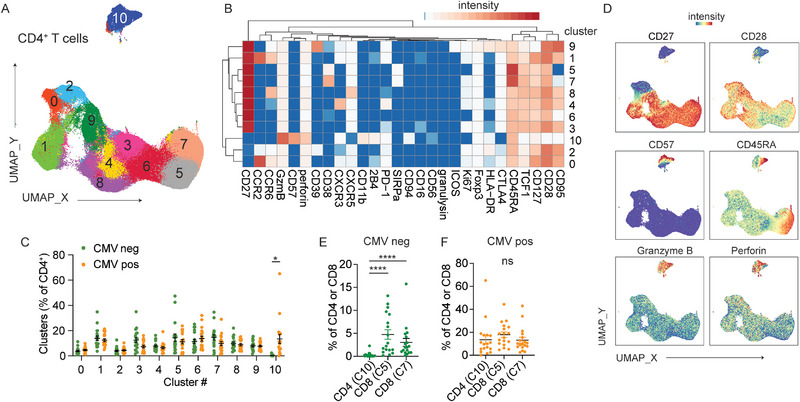
Expansion of terminally differentiated CD4⁺ T cell subsets in CMV‐seropositive individuals. (A) UMAP displays Phenograph‐defined clusters. Data combine 5000 manually gated CD19^−^CD3^+^TCRab^+^CD4^+^ cells from each donor (*n* = 36 donors). (B) The heatmap shows the median staining signal of individual markers for clusters shown in A. Markers used to select input cells were excluded. (C) Plot summarizes the percentage of CD4^+^ T cells in each cluster, divided by CMV serostatus (*n* = 18 per group). RM two‐way ANOVA with Sidak's multiple comparison test was performed. (D) UMAPs display the staining intensity of the indicated markers on CD4^+^ T cells. (E, F) Frequencies of CMV‐associated CD4⁺ T cell cluster (cluster 10) and CD8⁺ T cell clusters (clusters 5 and 7) are compared between individuals without (E) and with (F) CMV infection. Kruskal–Wallis test with Dun's multiple comparisons test was performed.

We performed mass cytometry with a 36‐marker panel using PBMCs from 20 RA patients and 16 controls with similar age and sex, with or without a positive CMV IgG antibody test (Table S1). PBMCs were barcoded using Cell‐ID, stained with metal‐conjugated antibodies focusing on T cell differentiation, and acquired together on the mass cytometer. Data normalization across runs was performed using a bead‐based standard [20]. Live CD3^+^TCRαβ^+^CD4^+^ T cells were identified by manual gating and combined for analyses using the Spectre pipeline (Figure S1A) [21]. Nonlinear dimensional reduction was performed using Uniform Manifold Approximation and Projection (UMAP) (Figure 1A). We observed two CD45RA^high^CD95^low^ naïve‐like T cell clusters (clusters 5 and 7), a CXCR5^+^ T follicular helper cell‐like cluster (cluster 8), and a CXCR3^+^ Th1‐like cluster (cluster 4). With respect to memory differentiation, clusters 0 and 2 lacked CD27 but retained CD28, whereas cluster 10 represented a more differentiated subset lacking both CD27 and CD28. This highly differentiated cluster was significantly expanded in CMV‐seropositive individuals and expressed cytotoxic molecules, including granzyme B (GzmB) and perforin (Figure 1B,C). Cells in cluster 10 also expressed higher levels of CD11b, 2B4, and CD94, markers commonly associated with NK cells and cytotoxic CD8^+^ T cells (Figure 1B). Additional markers of terminally differentiated CD4⁺ T cells, CD57 and CD45RA re‐expression, were also enriched in cluster 10, with heterogeneous and partially overlapping expression on the UMAP (Figure 1D). In the CD8⁺ compartment, CMV was associated with the expansion of two CD27^−^CD28^−^ CD8⁺ clusters (clusters 5 and 7, Figure S2). However, these terminally differentiated CD8⁺ subsets were less CMV‐restricted. Compared with cluster 10 of CD4^+^ T cells, they were present at higher frequencies in the absence of CMV infection (Figure 1E,F).

**FIGURE 2:**
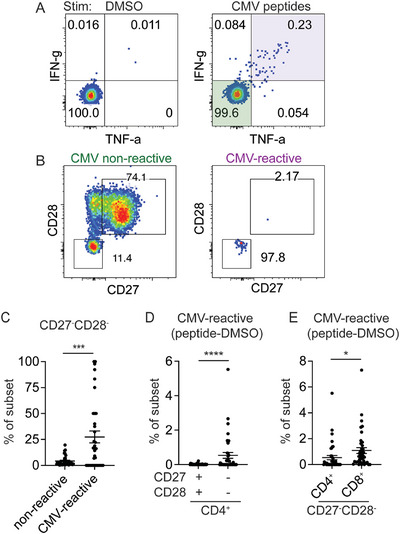
CMV‐reactive CD4⁺ T cells are enriched for the CD27^−^CD28^−^ phenotype. (A) Representative gating of CMV‐reactive CD4⁺ T cells. PBMCs from CMV‐seropositive individuals were stimulated with an overlapping CMV pp65 peptide pool. CMV‐reactive cells were identified by IFN‐γ and TNF‐α co‐expression. (B) Surface expression of CD27 and CD28 on TNF‐α^−^IFN‐γ^−^ (CMV nonreactive) and TNF‐α⁺IFN‐γ⁺ (CMV‐reactive) cells. (C) Plot summarizes the frequency of CD27^−^CD28^−^ phenotypic subset within CMV nonreactive and CMV‐reactive populations after peptide stimulation (*n* = 35). Samples without detectable TNF‐α⁺IFN‐γ⁺ response were removed. Wilcoxon matched‐pairs signed rank test was performed. (D) Frequency of TNF‐α⁺IFN‐γ⁺ cells as a percentage of CD27⁺CD28⁺ or CD27^−^CD28^−^ CD4⁺ subsets after DMSO background subtraction. Each dot represents a response from one donor (*n* = 40). Wilcoxon matched‐pairs signed rank test was performed. (E) The frequency of background‐subtracted CMV‐reactive T cells as a percentage of CD27^−^CD28^−^ CD4^+^ or CD27^−^CD28^−^ CD8^+^ T cells. The Wilcoxon matched‐pairs signed rank test was performed.

We confirmed the expansion of CD4⁺ cluster 10 in CMV‐seropositive donors by manual gating of CD27^−^CD28^−^ CD4⁺ T cells (Figure S1B). To determine whether CD27^−^CD28^−^ CD4⁺ T cell expansion in CMV infection was driven by CMV‐specific responses, we recruited additional CMV‐seropositive donors to focus on senescent CD4⁺ T cells (Tables S2 and S3). PBMCs from CMV‐seropositive donors were stimulated for 6 h with overlapping peptides from the major CD4⁺ T cell antigen pp65, followed by intracellular cytokine staining for TNF‐α and IFN‐γ. CMV‐reactive cells, identified by co‐expression of TNF‐α and IFN‐γ (Figure 2A), were enriched for CD27^−^CD28^−^ phenotype compared with nonreactive cells (Figure 2B,C). Consistent with this phenotypic skewing, CMV‐reactive T cells were more abundant in the CD27^−^CD28^−^ subset than in the CD27⁺CD28⁺ population (Figure 2D). Nonetheless, CMV‐reactive cells accounted for only a minor portion of the total CD27^−^CD28^−^ pool across both CD4⁺ and CD8⁺ compartments (Figure 2E), suggesting that CMV promotes broader bystander differentiation of the T cell repertoire beyond direct antigen‐specific responses.

### 2.2 The CD27^−^CD28^−^ CD4⁺ Population Encompasses Phenotypically Distinct Senescent Subsets

**FIGURE 3:**
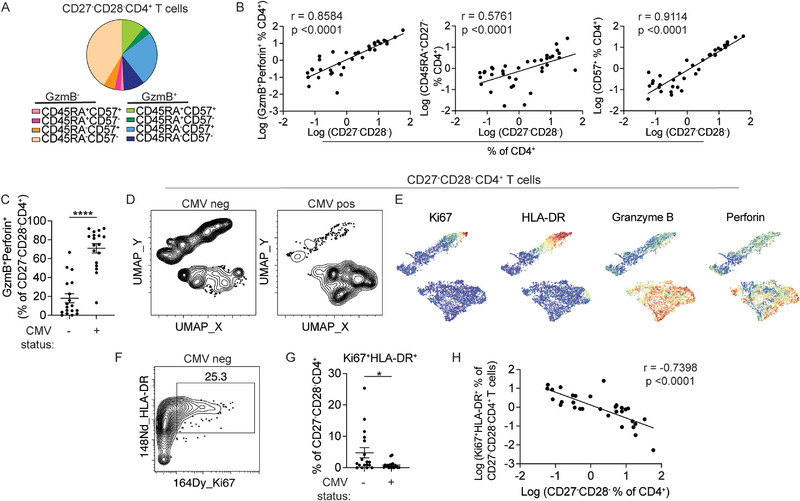
CD27^−^CD28^−^ CD4⁺ T cells display distinct phenotypes in CMV‐seropositive and seronegative individuals. (A) GzmB, CD45RA, and CD57 co‐expression in CD27^−^CD28^−^ CD4^+^ T cells. Marker combinations were determined using Boolean operators on manually defined gates. (B) The relationship between CD27^−^CD28^−^ CD4^+^ T cell frequency and cytotoxic (GzmB^+^Perforin^+^), TEMRA (CD45RA^+^CD27^−^), or CD57^+^ subsets. Pearson correlation was performed. (C) The frequency of GzmB^+^Perforin^+^ cells within the CD27^−^CD28^−^ CD4^+^ T cells in CMV seronegative and seropositive donors. Mann–Whitney test was performed. (D) UMAP distribution of CD27^−^CD28^−^ CD4^+^ T cells, separated by CMV status. (E) UMAPs display the staining intensity of the indicated markers. For (D) and (E), data combine comparable numbers of cells from CMV seropositive and seronegative individuals for a total of 6016 manually gated CD27^−^CD28^−^ CD4^+^ T cells. (F) Representative plot shows HLA‐DR, Ki67 staining in CD27^−^CD28^−^CD4^+^ T cells from a CMV seronegative individual. (G) Plot summarizes the frequencies of Ki67^+^HLA‐DR^+^ T cells as a percentage of CD27^−^CD28^−^ CD4^+^ subset in CMV‐seronegative or seropositive individuals. Mann–Whitney test was performed. (H) The correlation between CD27^−^CD28^−^ CD4^+^ T cell frequency and the abundance of Ki67^+^HLA‐DR^+^ cells within this subset. Each filled circle represents data from one individual (*n* = 36). Pearson correlation was performed. GzmB: granzyme B.

To further define the heterogeneity of CD27^−^CD28^−^ CD4⁺ T cells, we manually gated for CD57, CD45RA, and GzmB to quantify the distribution of these differentiation markers. Boolean analyses revealed that approximately half of the CD27^−^CD28^−^CD4^+^ T cells were GzmB positive. Among these, 73% co‐expressed CD57 and 22% expressed both CD57 and CD45RA. Within the GzmB negative subset, the majority were also negative for CD45RA and CD57 (Figure 3A). CD27^−^CD28^−^CD4^+^ T cell frequency correlated with increased cytotoxic granule content, TEMRA differentiation, and CD57 expression, which were all increased in CMV‐seropositive donors (Figure 3B; Figure S1C–E). Notably, the few CD27^−^CD28^−^CD4⁺ T cells from CMV‐seronegative individuals were phenotypically distinct and accounted for the majority of noncytotoxic CD27^−^CD28^−^ CD4⁺ T cells (Figure 3C). While an average of 71% of CD27^−^CD28^−^ subset from CMV seropositive individuals expressed GzmB and perforin, only 18% were positive for these cytotoxic molecules in CD27^−^CD28^−^CD4^+^ T cells from people without CMV infection. On the UMAP, CD27^−^CD28^−^ cells from CMV naïve donors clustered in a distinct region with low GzmB and perforin signal (Figure 3D,E). Instead, a portion of the CMV‐seronegative predominant region showed features of T cell activation by Ki67 and HLA‐DR expression (Figure 3E). Manually defined Ki67^+^HLA‐DR^+^ subsets were more abundant in donors without CMV infection and negatively correlated with CD27^−^CD28^−^CD4^+^ T cell expansion (Figure 3F–H).

**FIGURE 4:**
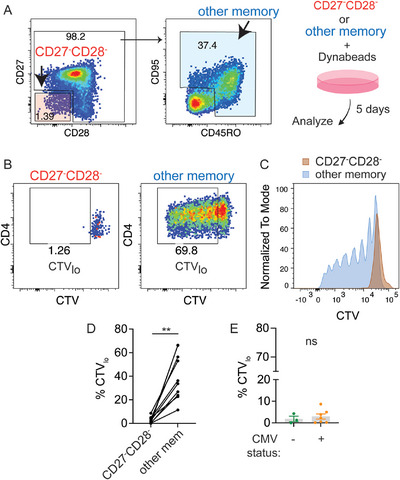
CD27^−^CD28^−^ CD4⁺ T cells show impaired proliferation independent of CMV status. (A) Representative plots show gating of CD27^−^CD28^−^ CD4^+^ T cells and non‐CD27^−^CD28^−^ memory cells (other memory). (B) CD27^−^CD28^−^ CD4^+^ T cells and other memory cells were sorted for stimulation with CD3/CD28 Dynabeads for 5 days. Plots show the percentage of the divided CTV low (CTV_lo_) subset. (C) The histogram shows the CTV profiles of CD27^−^CD28^−^ versus other memory CD4⁺ T cells. (D) Summary plot of the frequency of CTV_lo_ cells in CD27^−^CD28^−^ and other CD4^+^ memory subsets. Each filled circle represents data from one individual (*n* = 10). The Wilcoxon matched‐pairs signed rank test was performed. (E) Frequency of CTV_lo_ cells within CD27^−^CD28^−^ CD4^+^ subset after dynabead stimulation, grouped by CMV serostatus. Mann–Whitney test was used.

CD28^−^ CD4⁺ T cells are frequently classified as senescent due to reduced proliferative capacity [10]. CD27^−^CD28^−^ CD4⁺ T cells exhibited fewer features of terminal differentiation in CMV seronegative individuals, raising the possibility that they retain greater proliferative potential. To evaluate functional senescence, we sorted CD27^−^CD28^−^ CD4⁺ T cells from seven CMV‐seropositive and three CMV‐seronegative individuals and stimulated them with CD3/CD28 beads for 5 days. In parallel, non‐CD27^−^CD28^−^ memory T cells (other memory) from the same donors were sorted and stimulated as positive controls (Figure 4A; Figure S3A). Cell division was evaluated by CellTrace Violet (CTV) dilution. We found minimal CTV dilution of CD27^−^CD28^−^ CD4⁺ T cells after stimulation, confirming their replicative senescence. In contrast, matched memory cells from the same donors underwent robust proliferation (Figure 4B–D; Figure S3B). The extent of proliferative defect did not differ by CMV serostatus. Although CD27^−^CD28^−^ CD4⁺ T cells from CMV‐seronegative individuals displayed distinct phenotypic features, as shown in Figure 3, the frequency of dividing cells was similarly low and comparable to that observed in CMV‐seropositive donors (Figure 4E). These data demonstrate that CD27^−^CD28^−^ CD4⁺ T cells are functionally senescent in both CMV‐seropositive and seronegative individuals.

### 2.3 CMV Exerts a Dominant Effect on CD27^−^CD28^−^ CD4^+^ T Cell Expansion Over RA, EBV, and HSV

**FIGURE 5:**
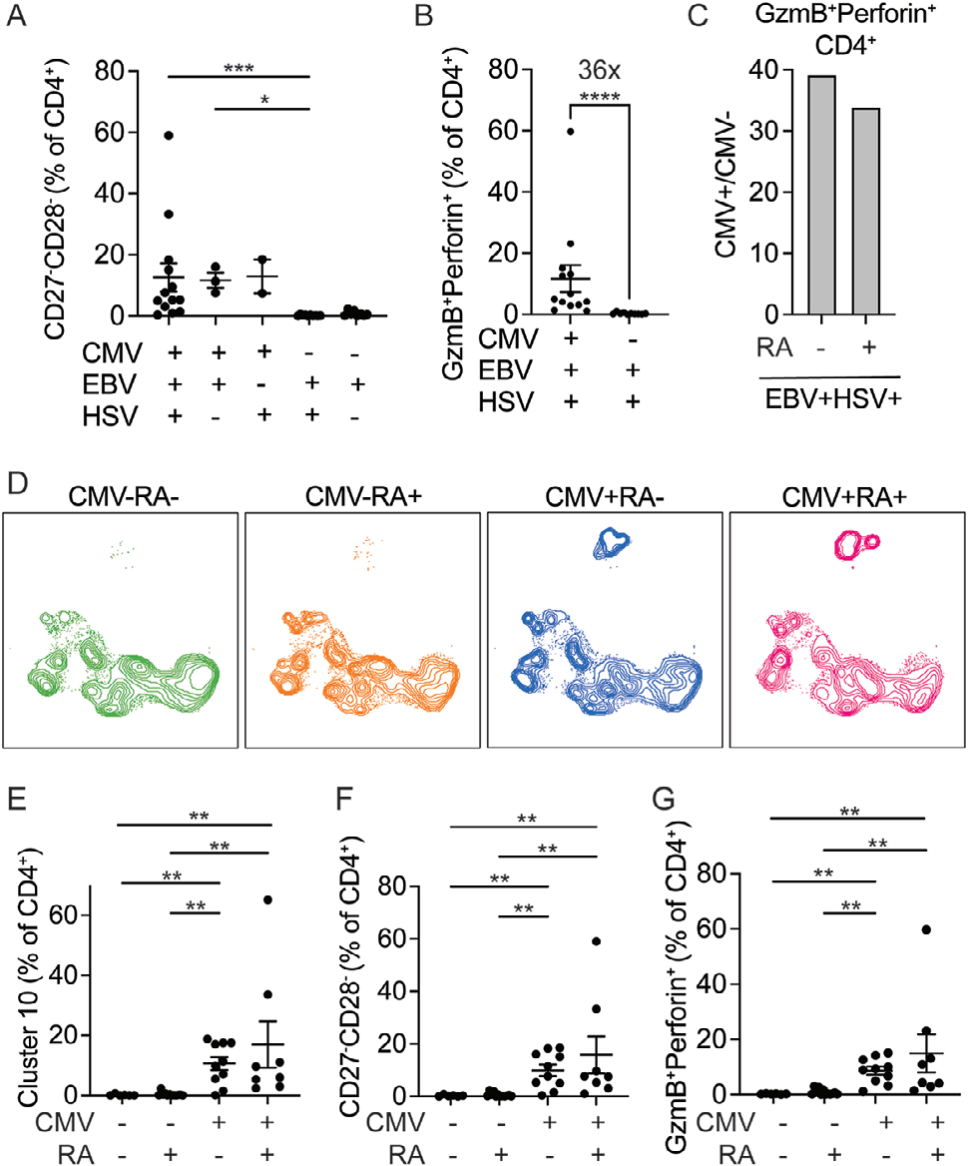
RA, EBV, and HSV have limited impact on senescent CD4⁺ T cell frequencies. (A) Frequencies of CD27^−^CD28^−^ subset as a percentage of CD4^+^ T cells, grouped by CMV, EBV, and HSV seropositivity. Each filled circle represents data from one individual (*n* = 36). Kruskal–Wallis and Dunn's multiple comparison tests were used. (B) Frequencies of GzmB and perforin‐expressing CD4^+^ T cells from EBV and HSV‐seropositive donors, with or without CMV co‐infection. Mann–Whitney test was performed. (C) The ratio of averaged GzmB^+^Perforin^+^ expression between CMV seropositive and seronegative individuals with EBV and HSV co‐infection, grouped by RA status. (D) UMAPs of CD27^−^CD28^−^CD4^+^ T cells, separated by CMV serostatus and RA diagnosis. CMV‐RA‐ (CMV seronegative controls), CMV‐RA+ (CMV seronegative RA patients), CMV+RA‐ (CMV seropositive controls), CMV+RA+ (CMV seropositive RA patients). (E–G) Plots summarize the percentage of CD4^+^ T cells in cluster 10 (E), negative for CD27 and CD28 (F), or expressing GzmB and perforin (G) by CMV and RA status. Kruskal‐Wallis and Dunn's multiple comparison tests were performed. GzmB: granzyme B.

In addition to CMV, other latent herpesviruses such as Epstein–Barr virus (EBV) and herpes simplex virus (HSV) are highly prevalent, and chronic inflammatory diseases like rheumatoid arthritis (RA) may further modulate immune aging and T cell differentiation. To evaluate the relative contributions of these persistent viral infections and RA, we performed serologic typing for EBV and HSV and grouped individuals by viral serostatus and RA diagnosis. We found that seropositivity for EBV and/or HSV in the absence of CMV co‐infection was not associated with increased frequencies of CD27^−^CD28^−^ CD4⁺ T cells (Figure 5A). Similarly, GzmB⁺Perforin⁺ cytotoxic CD4⁺ T cells remained low in EBV/HSV‐seropositive individuals lacking CMV, irrespective of RA status. CMV co‐infection was associated with a robust, 36‐fold expansion of GzmB⁺Perforin⁺ CD4⁺ T cells among EBV/HSV‐seropositive individuals (Figure 5B), with comparable frequencies observed in both RA patients and controls (Figure 5C). To further evaluate whether RA independently influences this population, individuals were stratified by CMV serostatus and RA diagnosis. As evident visually on UMAP, RA has minimal impact on the clustering distribution of CD27^−^CD28^−^ CD4⁺ T cells (Figure 5D). Frequencies of cluster 10, as well as manually gated CD27^−^CD28^−^ and GzmB⁺Perforin⁺ CD4⁺ T cells, were determined by CMV serostatus rather than RA (Figure 5E–G). Together, these findings identify CMV as the dominant factor driving the expansion of cytotoxic, senescent CD4⁺ T cells, surpassing the effects of other herpesviruses and RA.

### 2.4 CD27^−^CD28^−^ CD4^+^ T Cells Show Impaired Function in RA Patients

**FIGURE 6:**
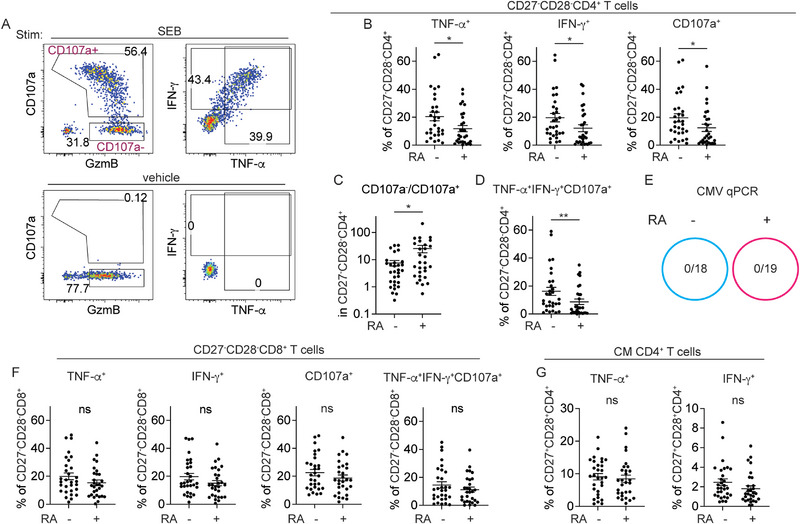
Senescent CD4^+^ T cells display decreased functional potential in RA patients. (A) PBMCs from 30 CMV‐seropositive healthy controls and 29 CMV‐seropositive RA patients were stimulated with SEB for 6 h and analyzed for cytokine production and degranulation. Representative flow plots show gating for the indicated markers under vehicle control and SEB‐stimulated conditions. (B) Frequencies of TNF‐α⁺, IFN‐γ⁺, and CD107a⁺ cells within CD27^−^CD28^−^ CD4⁺ T cells in RA patients and controls. (C) Ratio of CD107a^−^ to CD107a⁺ cells within the CD27^−^CD28^−^ CD4⁺ T cell subset. (D) Frequency of polyfunctional CD27^−^CD28^−^ CD4⁺ T cells co‐expressing TNF‐α, IFN‐γ, and CD107a, grouped by RA status. (E) CMV‐seropositive individuals, with or without RA, were analyzed for CMV DNA by quantitative PCR (qPCR). The ratio indicates the number of CMV DNA‐positive cases over the total tested. No individuals had detectable CMV DNA. (F) Frequencies of TNF‐α⁺, IFN‐γ⁺, CD107a⁺, and polyfunctional (TNF‐α⁺IFN‐γ⁺CD107a⁺) cells within CD27^−^CD28^−^ CD8⁺ T cells after stimulation. (G) Frequencies of TNF‐α⁺ and IFN‐γ⁺‐expressing cells within CD27⁺CD28⁺ central memory (CM) CD4⁺ T cells. Each filled circle represents data from one individual. Mann–Whitney test was performed.

Although we did not find a higher frequency of senescent CD27^−^CD28^−^CD4^+^ T cells in RA patients, RA and other disease‐associated factors may influence their function. To test this, PBMCs from 30 CMV‐seropositive controls and 29 CMV‐seropositive RA patients were stimulated with Staphylococcal Enterotoxin B (SEB) for 6 h and analyzed for production of cytokines, IFN‐γ and TNF‐α (Table S2 and 3; Figure 6A). Cytotoxic granule release was evaluated using surface expression of CD107a as a marker for degranulation (Figure 6A). Consistent with CyTOF data, the frequencies of CD27^−^CD28^−^ CD4⁺ T cells were not significantly elevated in CMV‐seropositive RA patients compared with CMV‐seropositive non‐RA controls (Figure S4A,B). However, CD27^−^CD28^−^ CD4⁺ T cells from these RA patients exhibited impaired function, producing significantly lower levels of TNF‐α and IFN‐γ (Figure 6B). Cells from RA patients also showed reduced CD107a expression, while GzmB expression remained similar between RA and control (Figure 6B; Figure S4C,D). The higher CD107a^−^/CD107a^+^ ratio in RA patients, representing the ratio of nondegranulating GzmB^+^ cells to the degranulating subset, suggests a defect in the release of cytotoxic granules by CD27^−^CD28^−^ CD4^+^ T cells (Figure 6C). Overall, CD27^−^CD28^−^ CD4⁺ T cells from RA patients were less polyfunctional, exhibiting a significantly lower frequency of cells co‐expressing TNF‐α, IFN‐γ, and CD107a (Figure 6D). The reduced frequency of TNF‐α⁺IFN‐γ⁺CD107a⁺ cells and other functional parameters in RA patients persisted after adjusting for age and sex. Among RA patients, stratification by sex and autoantibody profile also did not reveal significant differences in TNF‐α⁺IFN‐γ⁺CD107a⁺ frequencies within CD27^−^CD28^−^ CD4⁺ T cells (Figure S4E,F). Sex and age‐adjusted frequencies of TNF‐α⁺, IFN‐γ⁺, CD107a⁺, and TNF‐α⁺IFN‐γ⁺CD107a⁺ CD4⁺ T cells were not associated with other clinical characteristics, including anti‐citrullinated protein antibody (ACPA) status, erosive disease, smoking, or medication use (Table S4). Reflecting the absence of a clear treatment effect, RA patients with very low frequencies of TNF‐α⁺IFN‐γ⁺CD107a⁺ CD27^−^CD28^−^ CD4⁺ T cells (<1%) showed wide treatment heterogeneity and included untreated newly diagnosed individuals (Figure S5).

To assess whether functional impairment of CD27^−^CD28^−^ CD4⁺ T cells compromises CMV control, plasma CMV DNA level was measured using clinical quantitative PCR at the University of Pennsylvania Precision and Computational Diagnostics Laboratory. No CMV reactivation was detected, suggesting viral control is maintained, potentially through compensatory activity of other immune subsets (Figure 6E). Unlike CD27^−^CD28^−^ CD4⁺ T cells, the frequencies of TNF‐α⁺, IFN‐γ⁺, CD107a⁺, and polyfunctional TNF‐α⁺IFN‐γ⁺CD107a⁺ cells within the CD27^−^CD28^−^ CD8⁺ T cell compartments were not significantly diminished in RA patients (Figure 6F). Cytokine production by central memory (CD27⁺CD28⁺) CD4⁺ T cells was also not significantly altered in RA (Figure 6G). These findings delineate a selective functional defect among CD27^−^CD28^−^ CD4⁺ T cells in RA, highlighting disease‐associated dysregulation of terminally differentiated CD4^+^ T cells despite preserved responses in other memory and cytotoxic T cell subsets.

## 3 Discussion

Chronic antigen exposure drives CD4⁺ T cell senescence, but how distinct and overlapping stimuli shape T cell states remains unclear. CMV infection, a common latent herpesvirus carried by approximately 50% of adults in the United States [22, 23, 24], persists lifelong and promotes T cell terminal differentiation [9, 25]. Here, we examined the individual and combined contributions of chronic autoimmunity and latent viral infection by high‐dimensional immunophenotyping of CD4⁺ T cells in a cohort of RA patients and controls with known CMV serostatus.

We first evaluated the expression of canonical markers associated with late‐differentiated CD4⁺ T cell subsets to define senescence‐related phenotypes. This showed that CD57 is expressed by a restricted subset of CD28^−^ cells, partially overlapping with TEMRA cells that re‐expressed CD45RA. Cytotoxic features within the CD27^−^CD28^−^ subset were primarily observed in donors exposed to CMV. In the absence of CMV infection, CD27^−^CD28^−^ CD4⁺ T cells were still detectable, indicating that non‐CMV exposures and/or host‐intrinsic factors contribute to their differentiation. However, in CMV‐seronegative donors, CD27^−^CD28^−^ CD4⁺ T cells were infrequent and exhibited limited cytotoxic differentiation. Despite their phenotypic differences, those generated independent of CMV showed similarly impaired proliferative capacity in vitro compared with cells from CMV‐seropositive individuals, suggesting early emergence of proliferative defects. RA, EBV, and HSV were insufficient to drive a significant expansion of senescent CD4^+^ T cells in the absence of CMV, highlighting CMV's key role in promoting terminal CD4^+^ T cell differentiation. Notably, these results contrast with prior reports describing a RA‐associated accumulation of senescent CD4^+^ T cells [1, 2, 3]. The differences are not due to marker choice, as our findings remained consistent across multiple definitions of terminally differentiated CD4⁺ T cells. We speculate that prior associations with RA may reflect unaccounted effects of CMV. Supporting this, previous studies have shown that CD28^−^ CD4^+^ T cells are significantly more abundant in CMV‐seropositive than in CMV‐seronegative RA patients [26, 27]. In addition, other differences in study participants may contribute to the discrepancy. As most prior studies were conducted before or during the early years of biologic therapies, the impact of RA‐driven inflammation may have evolved with different treatments and more effective disease control over time.

Beyond frequency, the functional quality of CD27^−^CD28^−^ CD4⁺ T cells is an important consideration. Our data demonstrate that their functions are tunable and can be modulated by factors other than CMV. In RA, senescent CD4⁺ T cells were not more abundant after accounting for CMV status, but they displayed altered functional responses. We showed that CD27^−^CD28^−^ CD4⁺ T cells from CMV‐seropositive RA patients produced less IFN‐γ and TNF‐α and displayed reduced cytotoxic degranulation upon stimulation compared with cells from CMV‐seropositive individuals without RA. RA itself likely contributes to these findings. A highly impaired functional response could already be observed in two not yet treated RA patients in our cohort. While we did not identify a statistically significant association with medication usage, a potential influence cannot be excluded, as the lack of an association may reflect heterogeneous drug effects in a limited sample size. Effector responses in RA patients were more impaired in senescent CD4⁺ T cells compared with CD27^−^CD28^−^ CD8⁺ T cells and central memory CD4⁺ T cells, suggesting a selective functional vulnerability in this highly differentiated CD4⁺ subset. Given the absence of detectable CMV reactivation by quantitative PCR in these individuals, we speculate that compensatory immune mechanisms, potentially involving functionally preserved CD8⁺ T cells or other CD4⁺ subsets, may offset this dysfunction. However, such compensatory capacity is likely context‐dependent and may be insufficient under certain conditions. Factors such as aging or comorbid conditions could disrupt immune equilibrium in disease settings. Considering the role of cytotoxic CD4⁺ T cells in tumor surveillance, we suggest that suppression of senescent CD4⁺ T cells may contribute to the increased cancer risk in RA patients under permissive conditions [28, 29]. Future studies are needed to elucidate the mechanism by which RA influences senescent CD4^+^ T cells and the immunological consequences of these changes.

In conclusion, CMV drives senescent CD4⁺ T cell differentiation, while RA exerts a distinct, nonredundant modulatory effect that further shapes the functional capacity of terminally differentiated CD4⁺ T cells. These findings underscore the differential impacts of chronic inflammation and highlight the importance of accounting for both exposure history and host factors in future studies of human T cell differentiation.

### 3.1 Data Limitations and Perspectives

Although CD27^−^CD28^−^ CD4⁺ T cells showed minimal proliferation with CD3/CD28 stimulation, their proliferative capacity may differ under alternative conditions, such as IL‐15 and other cytokines, which were not assessed in this study. In evaluating the impact of RA, we examined only a subset of effector functions. CD27^−^CD28^−^ CD4⁺ T cells from RA patients may exhibit additional changes not captured in our analysis. Other limitations include the modest sample size of this study and the need to further investigate the mechanisms underlying the observed functional alterations in RA patients. Whether similar changes in senescent CD4⁺ T cells occur in other autoimmune diseases remains to be determined. Future studies examining broader functional, replicative, and transcriptional programs in CD27^−^CD28^−^ CD4⁺ T cells will be essential to define the role of senescent CD4⁺ T cells in autoimmunity and chronic infection, and to inform strategies for modulating their activity.

## 4 Methods and Methods

### 4.1 Sex as a Biological Variable

Both male and female participants were included in this study. Sex was analyzed as a biological variable.

### 4.2 Human Samples

Blood samples were collected from healthy volunteers and rheumatology clinic patients at the University of Pennsylvania and Corporal Michael J Crescenz VA Medical Center from 2015 to 2023. All samples were de‐identified and obtained with IRB regulatory approval from the University of Pennsylvania or Corporal Michael J Crescenz VA Medical Center. Informed consent was obtained from all participants. For sample processing, Vacutainer tubes were spun down for plasma collection. PBMCs were isolated by density gradient centrifugation (Ficoll‐Paque, GE Healthcare) and cryopreserved in fetal bovine serum (FBS) with 10% DMSO. Subject characteristics are shown in Tables S1 and S2. The high proportion of males in the CyTOF cohort reflects recruitment from the VA, which has a predominantly male patient population.

### 4.3 CMV, HSV, and EBV Serologic Testing

CMV, HSV, and EBV ELISA on plasma samples were performed and interpreted according to the manufacturer's instructions. Manufacturer‐defined positivity index was used. Samples with ambiguous results were retyped and excluded if the result remained equivocal after three repeats. CMV typing was performed using the Cytomegalovirus IgG ELISA Kit (KA1452, Abnova). HSV typing was performed using Simplex Virus I IgG ELISA Kit (KA0229, Abnova). EBV typing was performed using Epstein–Barr Virus EBNA‐1 IgG ELISA Kit (KA1448, Abnova).

### 4.4 CyTOF Staining and Analyses

#### 4.4.1 CyTOF Staining

Cryopreserved PBMCs were thawed, washed, and incubated with anti‐CD127, anti‐CCR2, anti‐CD27, anti‐CXCR3, anti‐CCR6, and anti‐TCRαβ for 30 min at room temperature before cisplatin staining, fixation, and cell ID barcoding according manufacturer's protocol (StandardBioTools). Metal barcoded cells were pooled into a single tube and stained with the remainder of surface antibodies for 30 min at room temperature. For intracellular staining, cells were permeabilized and fixed using Foxp3 staining buffer set (eBioscience) and incubated with the intracellular antibody cocktail for 1 h at room temperature (Table S5). Metal conjugation of CyTOF antibodies was performed according to the manufacturer's protocol using the X8 Maxpar kit (StandardBioTools). After staining, cells were washed three times and then resuspended in 2% paraformaldehyde (Electron Microscopy Sciences) with 125 nM iridium intercalator (StandardBioTools) for an overnight incubation at 4⁰C. The next day, cells were washed three times, including a final wash in distilled water, and resuspended in water containing normalization beads before acquisition on Helios. Bead standards were used to normalize CyTOF runs with the Matlab‐based Nolan lab normalizer [20].

#### 4.4.2 Data Analyses

Doublets and beads were excluded from Iridium^+^Cisplatin^−^ cells. From each sample, equal numbers of manually gated CD19^−^CD3^+^TCRαβ^+^CD4^+^ cells were downsampled and exported using FlowJo (BD Biosciences). For CD4^+^ T cell analyses, a total of 180,000 cells were read into R by flowCore and combined into a single dataset for subsequent data processing and high‐dimensional analyses using the Spectre package in R [21]. Staining intensities were converted using the Arcsinh transformation with a cofactor of 5. Clustering was performed using Phenograph with nearest neighbors set to 1100 (*k* = 1100) [30]. Unbiased UMAP was used for dimensional reduction and visualization [31]. The color scale was modified to use the same color for 0 and values under 0 after the Arcsinh transformation. The heatmap was generated using the “gplot” package in R and showed the raw staining intensity of each marker after arcsinh transformation. Non‐T cell markers and those used to select input cells were excluded. Heatmap dendrograms were clustered by Euclidean distance. CD27^−^CD28^−^CD4^+^ T cell analyses were performed as above using 6016 manually gated CD27^−^CD28^−^ population with a cofactor of 5 and *k* = 250.

### 4.5 T Cell Stimulation Assays

#### 4.5.1 Intracellular Cytokine Staining

For polyclonal stimulation, cryopreserved PBMCs were thawed, rested overnight, and plated at 300,000 to 1 million cells per well in 200 µL RPMI medium in U‐bottom 96‐well plates. Cells were incubated with SEB (1 µg/mL, Toxin Technology) or vehicle control (DMSO) for 6 h in the presence of monensin (2 µM, Sigma), brefeldin A (5 µg/mL, Sigma), and anti‐CD107a antibody (BioLegend). To identify CMV‐reactive T cells, PBMCs were stimulated overnight with 15‐mer peptides with 11‐amino acid overlap spanning CMV pp65 at 0.5–1 ng/µL (JPT). Monensin, brefeldin A, and anti‐CD107a antibody were added during the final 6 h of incubation. Following stimulation, cells were washed and stained with viability dye and surface antibodies, including CD27 and CD28, for 30 min at room temperature. Intracellular staining for TNF‐α, IFN‐γ, granzyme B, perforin, CD3, CD4, and CD8 (Table S6) was performed using the BD Cytofix/Cytoperm fixation/permeabilization kit according to the manufacturer's instructions. After staining, cells were fixed with 2% paraformaldehyde, acquired using an LSRII flow cytometer (BD), and the data were analyzed with FlowJo software (BD). For CMV‐reactive T cells, peptide‐stimulated responses were background‐subtracted from the DMSO control, with negative values set to a minimum of zero.

#### 4.5.2 Proliferation

Cryopreserved T cells were thawed, rested for 1 h, and labeled with CellTrace Violet (CTV) dye according to the manufacturer's instructions (Invitrogen). CTV‐labeled cells were then stained with viability dye and surface antibodies against CD3, CD4, CD27, CD28, CD95, and CD45RO (Table S6) for 30 min at room temperature. CD27^−^CD28^−^ and non‐CD27^−^CD28^−^ memory (other memory) CD4⁺ T cells were sorted using a FACSAria (BD) cell sorter, following the gating strategy shown in Figure S3A. Equal numbers of CD27^−^CD28^−^ and other memory CD4⁺ T cells from the same donors were stimulated with CD3/CD28 Dynabeads at a 1:1 bead‐to‐cell ratio and cultured for 5 days. Cells were harvested, stained with viability dye and antibodies against CD3 and CD4 for 30 min at 4°C, acquired on an LSRII flow cytometer (BD), and analyzed using FlowJo software (BD).

### 4.6 Statistics

Normality was assessed using the Shapiro–Wilk test. A nonparametric test was used if any of the variables were non‐normally distributed. Otherwise, a parametric test was used. For Pearson or Spearman correlations, least squares linear regression was used to calculate the best‐fitting line. Statistical comparisons were performed using Welch's *t*‐test, Mann–Whitney test, Wilcoxon matched‐pairs signed rank test, and repeated measures (RM)‐two‐way ANOVA. Associations with RA disease characteristics were assessed using univariate linear regression. A *p*‐value of <0.05 was used as the significance level and adjusted if multiple comparisons were performed. Statistical analyses were performed using GraphPad Prism. Lines and bars represent the mean, and variability is represented by the standard error of the mean (SEM). *p < 0.05, **p < 0.01, ***p < 0.001, ****p < 0.0001.

### 4.7 Study Approval

All participants have given written informed consent. The study was approved by the Institutional Review Board at the University of Pennsylvania, Philadelphia (approval 819711, 820884) and Corporal Michael J Crescenz VA Medical Center (approval 01480).

## Author Contributions

Conceptualization: Laura F. Su; Experimentation: Lea Williams, Ali O. Saber, Asgar Ansari, Xi Su, Laura F. Su; High‐dimensional phenotypic analyses: Ruozhang Xu, Ali O. Saber; Study recruitment: Hannah Jung, Silina Awad, Anupama Shahane; Modeling and statistical support: Joshua F. Baker; Supervision: Laura F. Su; Manuscript preparation: Lea Williams, Ali O. Saber, Joshua F. Baker, Laura F. Su. Authors’ order reflects relative contribution.

## Conflicts of Interest

The authors declare no conflicts of interest.

## Peer Review

The peer review history for this article is available at https://doi.org/10.1002/eji.70093.

## Supporting information




**Supporting File**: eji70093‐sup‐0001‐SuppMat.docx.

## Data Availability

All data needed to evaluate the conclusions in the paper are present in the paper or the supplementary materials. Analyses are performed using standard analysis packages. Further information is available from the corresponding author upon reasonable request.
