# Supplementary material for: Divergent Effects of Cytomegalovirus and Rheumatoid Arthritis on Senescent CD4+ T Cells
Source: Eur J Immunol. 2025 Nov 14;55(11):e70093. doi: 10.1002/eji.70093 (PMC12616766; doi:10.1002/eji.70093)
Supplement: Supplementary file 1 — Supporting File: eji70093‐sup‐0001‐SuppMat.docx. [file EJI-55-e70093-s001.docx]

**Supplementary Material**

**
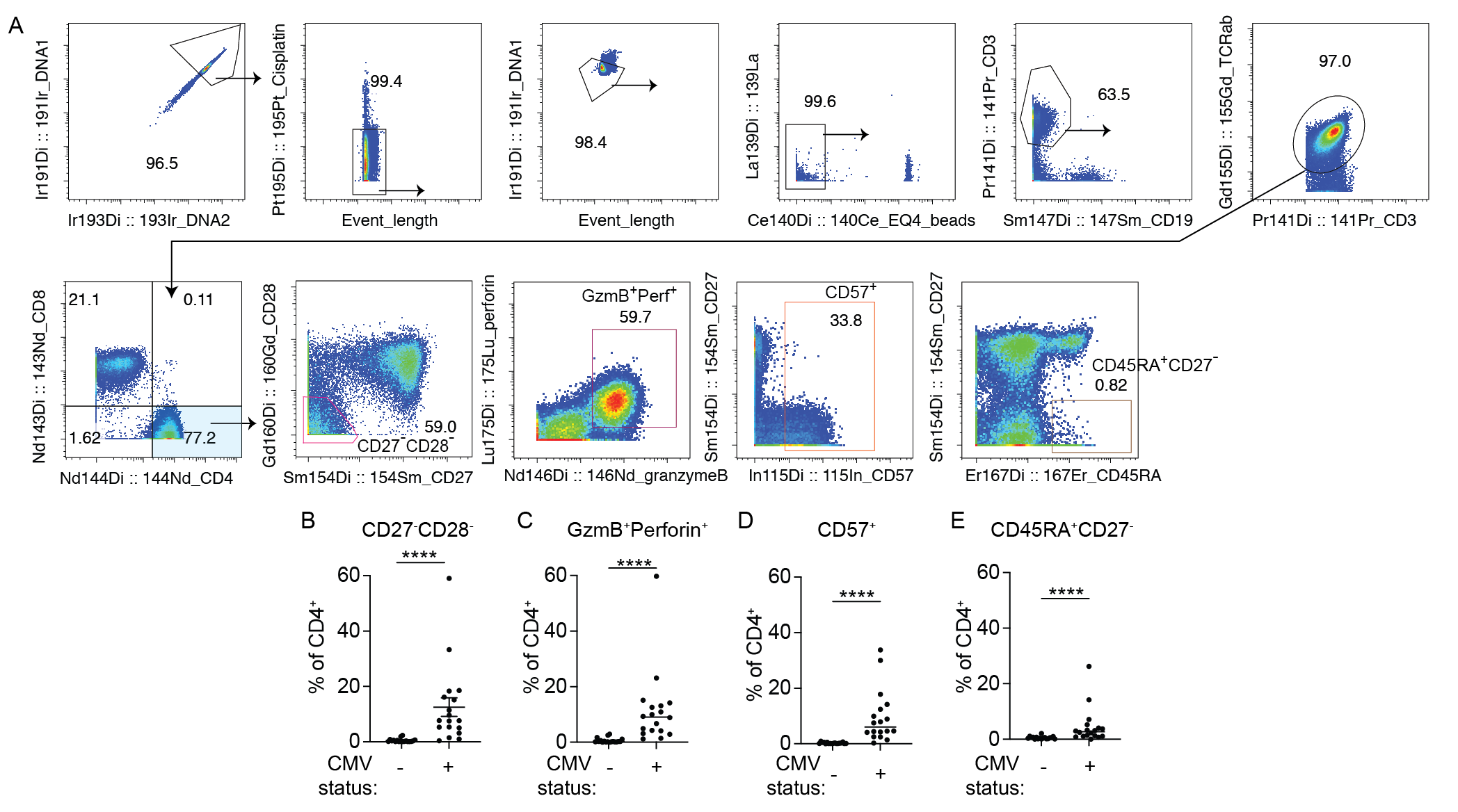
**

**Figure S1: Identification of various differentiated CD4^+^ T cell subsets by mass cytometry.**

(A) Representative plots show the gating strategy for identifying total CD4^+^ T cells and CD27^-^CD28^-^, GzmB^+^Perforin^+^, CD57^+^, CD45RA^+^CD27^-^ subsets in the PBMCs from a CMV-seropositive individual. (B-E) The frequency of the indicated subset as a percentage of CD4^+^ T cells in CMV seronegative and seropositive donors. Each symbol represents cells from one individual. Mann-Whitney test was performed.


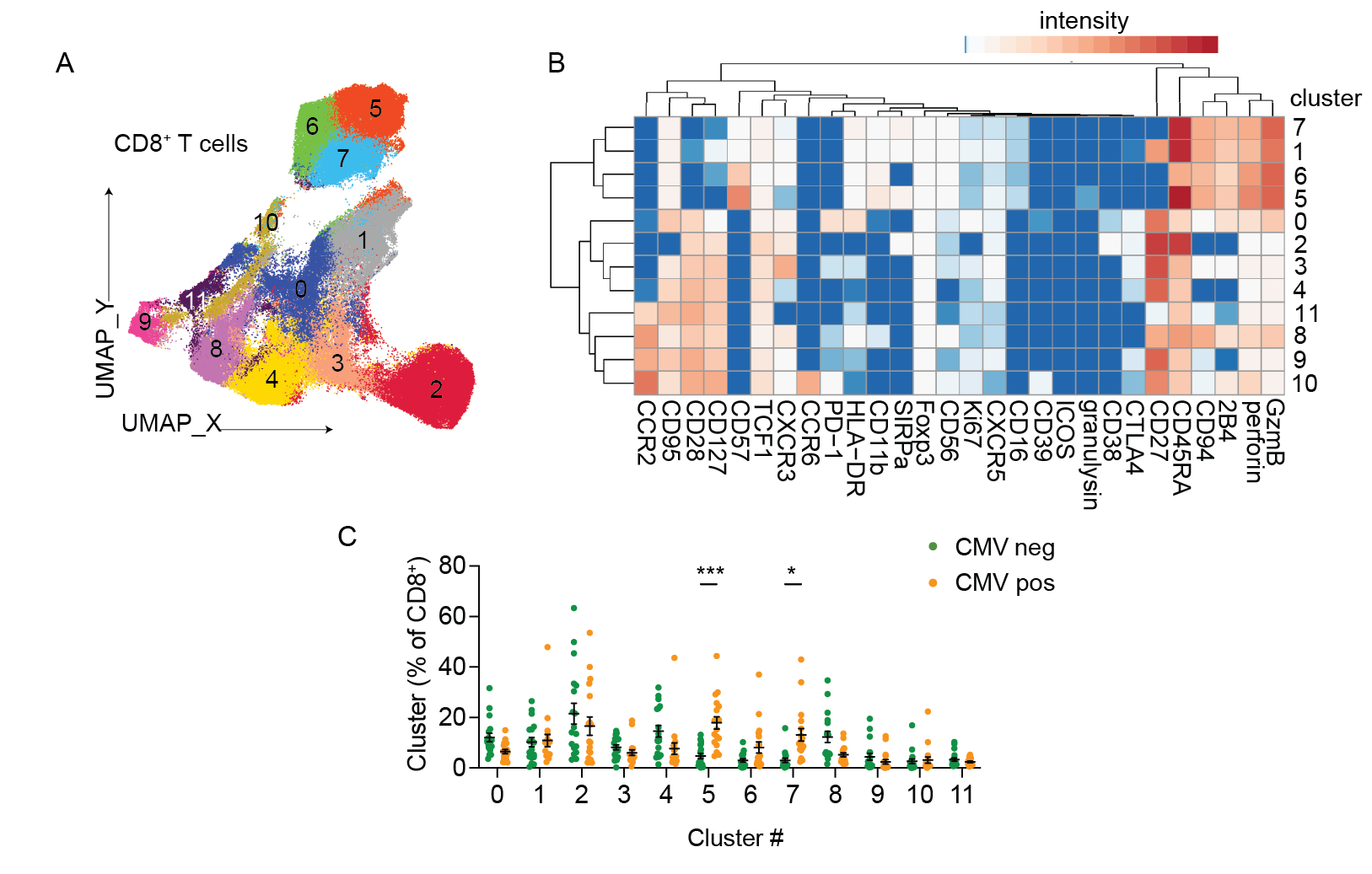


**Figure S2: Altered CD8⁺ T cell composition in CMV-seropositive individuals.**

(A) UMAP displays Phenograph-defined clusters. Data combine 5,000 manually gated CD19^-^CD3^+^TCRab^+^CD8^+^ cells from each donor (n = 36 donors). (B) Heatmap shows the median staining signal of individual markers for clusters shown in A. Markers used to select input cells were excluded. (C) Plot summarizes the percentage of CD8^+^ T cells in each cluster, divided by CMV serostatus (n = 18 per group). RM two-way ANOVA with Sidak’s multiple comparison test was performed.


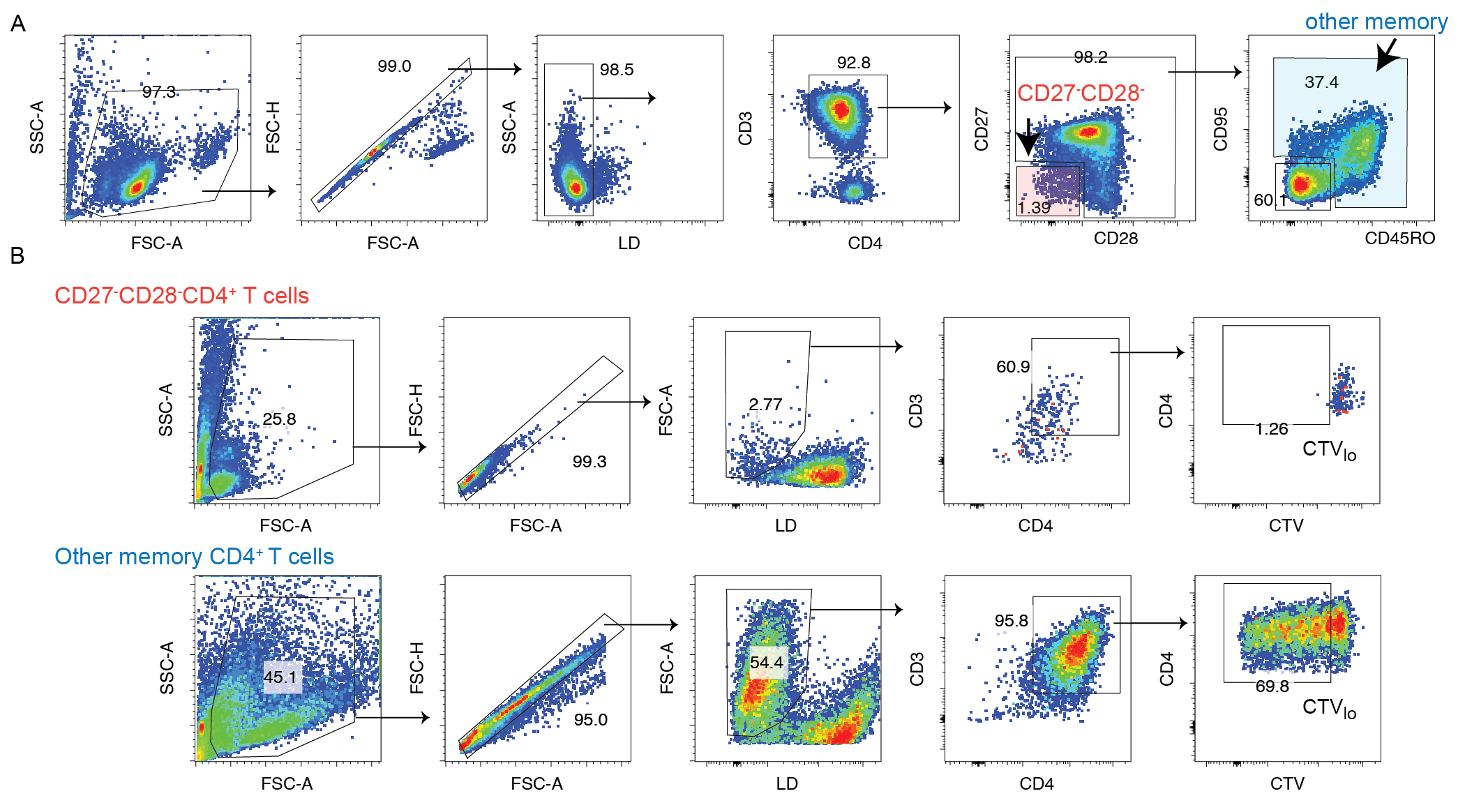


**Figure S3: Analyses of T cell proliferation.**

(A) CD27⁻CD28⁻ and non-CD27⁻CD28⁻ memory CD4⁺ T cells were sorted and stimulated with CD3/CD28 Dynabeads for 5 days. Representative plots show the gating strategy used for sorting both subsets. (B) Plots show the gating strategy used to identify proliferating CD4^+^ T cells based on CellTrace violet (CTV) staining after 5 days in culture.

**
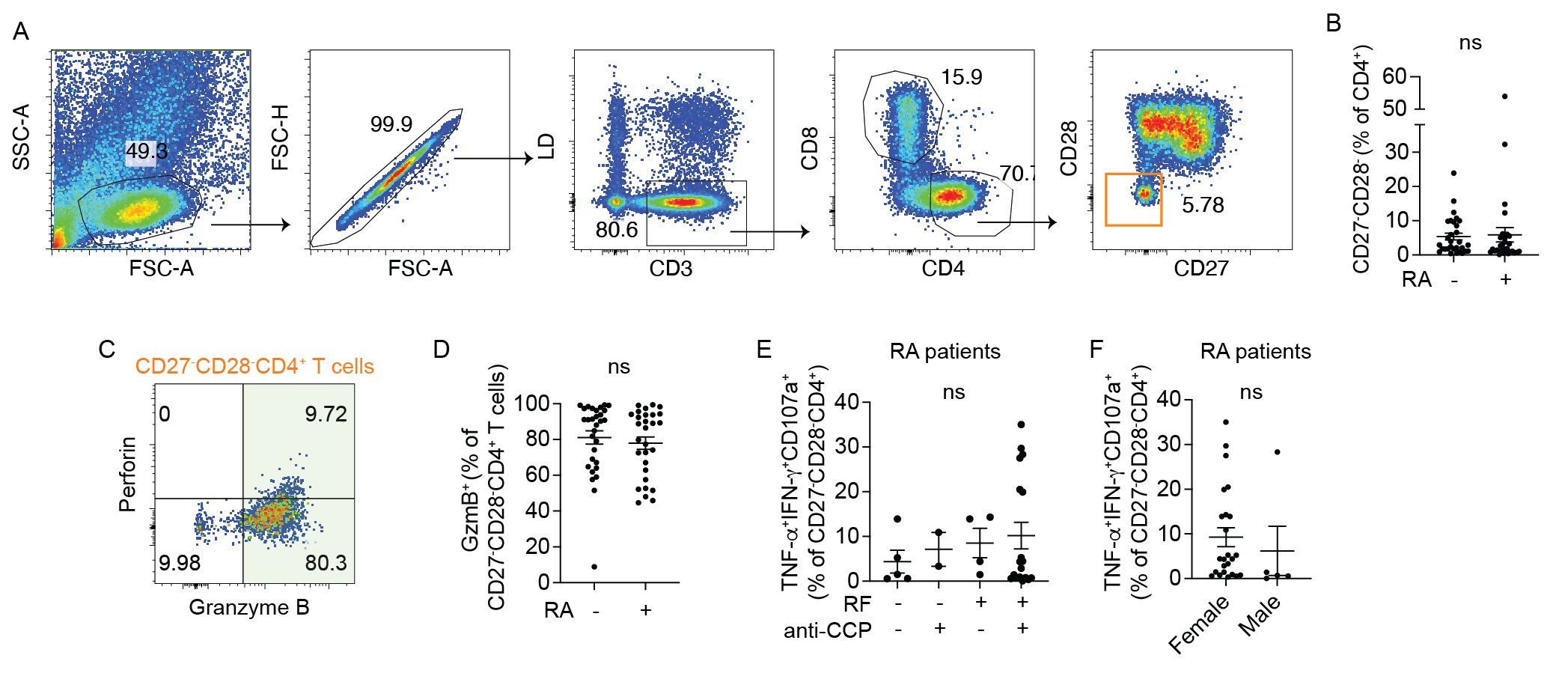
**

**Figure S4: Functional responses of CD27^-^CD28^-^** **CD4^+^ T cells to SEB stimulation.** (A) Representative gating strategy for identifying CD27^-^CD28^-^ CD4^+^ T cells in SEB stimulation assay. (B) Summary plot showing the frequency of CD27⁻CD28⁻ CD4⁺ T cells in RA patients and healthy controls. Statistical comparison was performed using the Mann–Whitney test. (C) Representative plot showing perforin and GzmB expression in the DMSO-treated condition. (D) Summary plot shows the frequency of total GzmB⁺ cells (colored box in C) in RA patients and controls. Mann-Whitney test was performed. (E) Frequency of TNF-α, IFN-γ, and CD107a-expressing CD27⁻CD28⁻ CD4⁺ T cells by autoantibody status in RA patients. Kruskal-Wallis test and Dunn’s multiple comparisons test were performed. (F) Frequency of TNF-α, IFN-γ, and CD107a-expressing CD27⁻CD28⁻ CD4⁺ T cells in RA patients, grouped by sex. Mann-Whitney test was performed. RF: rheumatoid factor, anti-CCP: anti-cyclic citrullinated peptide antibody.


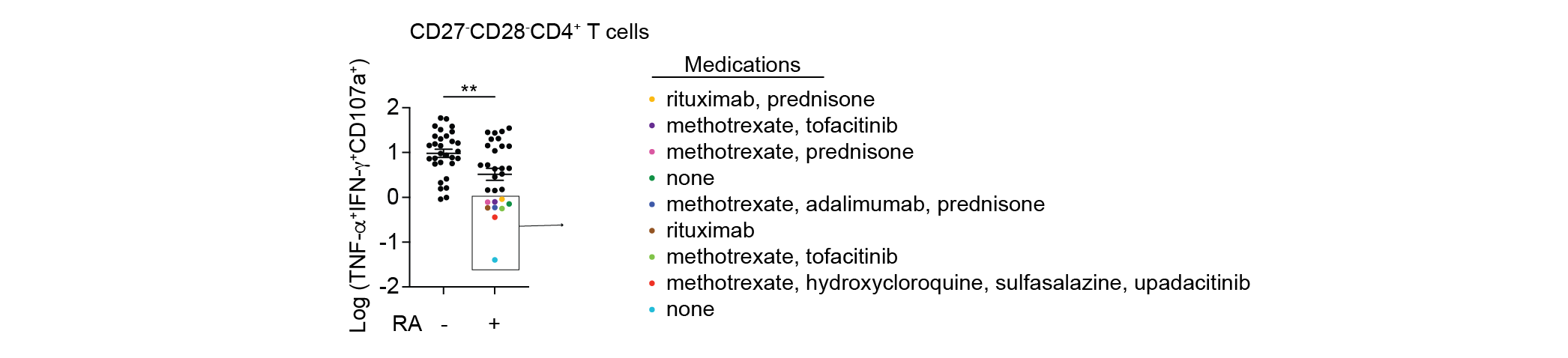


**Figure S5: Medications taken by RA patients with severely reduced CD27^-^CD28^-^CD4^+^ T cell function.**

Box highlights RA patients with fewer than 1% of TNF-α^+^IFN-γ^+^CD107^+^ cells within the CD27^-^CD28^-^CD4^+^ T cell subset. Medications taken by each RA patient are color-coded as indicated. Welch’s t-test was performed.

**Table S1:** **CyTOF cohort participant characteristics**

|  | CMV+RA- (n = 10) | CMV-RA- (n = 6) | CMV+RA+ (n = 8) | CMV-RA+ (n = 12) |
| --- | --- | --- | --- | --- |
| Female – n (%) | 2 (20) | 1 (16.7) | 2 (25) | 4 (33.3) |
| Age – year (mean + SD) | 64.6 (12.1) | 63.6 (6.7) | 61.6 (8.7) | 61.6 (13.6) |
| Race: |  |  |  |  |
| White – n (%) | 5 (50) | 4 (66.7) | 3 (37.5) | 8 (66.7) |
| African American/Black - n (%) | 4 (40) | 2 (33.3) | 4 (50) | 4 (33.3) |
| Others – n (%) | 1 (10) | 0 | 1 (12.5) | 0 |
| Current smoker – n (%) | 5 (50) | 2 (33.3) | 2 (25) | 2 (16.7) |
| BMI – kg/m^2^ (mean + SD) | 31.6 (5.1) | 34.1 (6.5) | 30.5 (9.2) | 29.3 (8.4) |
| CMV seropositivity – n (%) | 10 (100) | 0 (0) | 8 (100) | 0 (0) |
| EBV seropositivity – n (%) | 9 (90) | 6 (100) | 7 (87.5) | 12 (100) |
| HSV seropositivity – n (%) | 7 (70) | 5 (83.3) | 8 (100) | 5 (41.7) |
| RF positive – n (%) | NA | NA | 8 (100) | 10 (83.3) |
| CCP positive – n (%) | NA | NA | 8 (100) | 9 (75) |
| Disease duration – years (mean + SD) | NA | NA | 11.7 (13.0) | 10.4 (11.2) |
| DAS28-CRP (mean + SD) | NA | NA | 3.4 (1.7) | 3.3 (1.7) |
| Medications: |  |  |  |  |
| Methotrexate – n (%) | NA | NA | 2 (25) | 8 (66.7) |
| Anti-TNFs – n (%) | NA | NA | 4 (50) | 1 (8.3) |
| Rituximab – n (%) | NA | NA | 1 (12.5) | 0 |
| Tofacitinib – n (%) | NA | NA | 0 | 3 (25) |
| Corticosteroids – n (%) | NA | NA | 2 (25) | 4 (33) |

NA: not applicable

**Table S2: FACS cohort participant characteristics**

|  | Control (n = 30) | RA (n = 29) |
| --- | --- | --- |
| Female – n (%) | 16 (53.3) | 24 (82.8) |
| Age – year (mean + SD) | 47.5 (18.0) | 53.8 (16.1) |
| Race: |  |  |
| White – n (%) | 13 (43.3) | 11 (37.9) |
| African American/Black – n (%) | 8 (26.7) | 14 (48.3) |
| Asian – n (%) | 4 (13.3) | 0 (0) |
| Others – n (%) | 5 (16.7) | 4 (13.8) |
| Current smoker – n (%) | 5 (16.7) | 7 (24.1) |
| BMI – kg/m2 (mean + SD) | 28.6 (7.9) | 29.4 (6.3) |
| CMV seropositivity – n (%) | 30 (100) | 29 (100) |
| RF positive – n (%) | NA | 21 (75.0) |
| CCP positive – n (%) | NA | 19 (67.9) |
| Disease duration – years (mean + SD) | NA | 8.0 (8.7) |
| DAS28-CRP (mean + SD) | NA | 3.5 (1.6) |
| Medications: |  |  |
| Methotrexate – n (%) | NA | 15 (51.7) |
| Anti-TNFs – n (%) | NA | 2 (6.9) |
| Tofacitinib or upadacitinib – n (%) | NA | 6 (20.7) |
| Rituximab – n (%) | NA | 2 (6.9) |
| Corticosteroids – n (%) | NA | 7 (24.1) |
| Untreated – n (%) | NA | 6 (20.7) |

NA: not applicable

**Table S3: Summary of donors across experimental assays**

| ID | RA | CMV | Experiments |  | ID | RA | CMV | Experiments |
| --- | --- | --- | --- | --- | --- | --- | --- | --- |
| C01 | No | - | CyTOF |  | RA01 | Yes | - | CyTOF |
| C02 | No | - | CyTOF |  | RA02 | Yes | - | CyTOF |
| C03 | No | - | CyTOF |  | RA03 | Yes | - | CyTOF |
| C04 | No | - | CyTOF |  | RA04 | Yes | - | CyTOF |
| C05 | No | - | CyTOF |  | RA05 | Yes | - | CyTOF |
| C06 | No | - | CyTOF |  | RA06 | Yes | - | CyTOF |
| C07 | No | + | CyTOF |  | RA07 | Yes | - | CyTOF |
| C08 | No | + | CyTOF, FACS ( SEB) |  | RA08 | Yes | - | CyTOF |
| C09 | No | + | CyTOF |  | RA09 | Yes | - | CyTOF |
| C10 | No | + | CyTOF |  | RA10 | Yes | - | CyTOF |
| C11 | No | + | CyTOF |  | RA11 | Yes | - | CyTOF |
| C12 | No | + | CyTOF |  | RA12 | Yes | - | CyTOF |
| C13 | No | + | CyTOF |  | RA13 | Yes | + | CyTOF |
| C14 | No | + | CyTOF, FACS ( SEB) |  | RA14 | Yes | + | CyTOF |
| C15 | No | + | CyTOF, FACS ( SEB) |  | RA15 | Yes | + | CyTOF |
| C16 | No | + | CyTOF |  | RA16 | Yes | + | CyTOF, FACS (SEB) |
| C17 | No | + | FACS (CMV peptides, SEB) |  | RA17 | Yes | + | CyTOF, FACS (SEB) |
| C18 | No | + | FACS (SEB) |  | RA18 | Yes | + | CyTOF |
| C19 | No | + | FACS (CMV peptides, SEB) |  | RA19 | Yes | + | CyTOF |
| C20 | No | + | FACS (SEB) |  | RA20 | Yes | + | CyTOF |
| C21 | No | + | FACS (SEB) |  | RA21 | Yes | + | FACS (CMV peptides, SEB) |
| C22 | No | + | FACS (SEB) |  | RA22 | Yes | + | FACS (CMV peptides, SEB) |
| C23 | No | + | FACS (CMV peptides, SEB), CTV |  | RA23 | Yes | + | FACS (CMV peptides, SEB) |
| C24 | No | + | FACS (CMV peptides, SEB), CTV |  | RA24 | Yes | + | FACS (CMV peptides, SEB) |
| C25 | No | + | FACS (CMV peptides, SEB) |  | RA25 | Yes | + | FACS (CMV peptides, SEB) |
| C26 | No | + | FACS (CMV peptides, SEB) |  | RA26 | Yes | + | FACS (CMV peptides, SEB) |
| C27 | No | + | FACS (SEB) |  | RA27 | Yes | + | FACS (CMV peptides, SEB) |
| C28 | No | + | FACS (SEB) |  | RA28 | Yes | + | FACS (CMV peptides, SEB) |
| C29 | No | + | FACS (SEB) |  | RA29 | Yes | + | FACS (SEB) |
| C30 | No | + | FACS (SEB) |  | RA30 | Yes | + | FACS (CMV peptides, SEB) |
| C31 | No | + | FACS (CMV peptides, SEB) |  | RA31 | Yes | + | FACS (CMV peptides, SEB) |
| C32 | No | + | FACS (CMV peptides, SEB) |  | RA32 | Yes | + | FACS (CMV peptides, SEB) |
| C33 | No | + | FACS (CMV peptides, SEB), CTV |  | RA33 | Yes | + | FACS (SEB) |
| C34 | No | + | FACS (CMV peptides, SEB) |  | RA34 | Yes | + | FACS (CMV peptides, SEB) |
| C35 | No | + | FACS (CMV peptides, SEB) |  | RA35 | Yes | + | FACS (CMV peptides, SEB) |
| C36 | No | + | FACS (CMV peptides, SEB) |  | RA36 | Yes | + | FACS (CMV peptides, SEB) |
| C37 | No | + | FACS (CMV peptides, SEB) |  | RA37 | Yes | + | FACS (CMV peptides, SEB) |
| C38 | No | + | FACS (CMV peptides, SEB) |  | RA38 | Yes | + | FACS (CMV peptides, SEB) |
| C39 | No | + | FACS (CMV peptides, SEB) |  | RA39 | Yes | + | FACS (CMV peptides, SEB) |
| C40 | No | + | FACS (CMV peptides, SEB) |  | RA40 | Yes | + | FACS (CMV peptides, SEB) |
| C41 | No | + | FACS (CMV peptides, SEB), CTV |  | RA41 | Yes | + | FACS (SEB) |
| C42 | No | + | FACS (CMV peptides, SEB), CTV |  | RA42 | Yes | + | FACS (CMV peptides, SEB) |
| C43 | No | + | FACS (CMV peptides, SEB), CTV |  | RA43 | Yes | + | FACS (SEB), CTV |
| C44 | No | - | CTV |  | RA44 | Yes | + | FACS (SEB) |
| C45 | No | - | CTV |  | RA45 | Yes | + | FACS (SEB) |
| C46 | No | - | CTV |  | RA46 | Yes | + | FACS (CMV peptides, SEB) |
|  |  |  |  |  | RA47 | Yes | + | FACS (CMV peptides, SEB) |

**Table S4:** Associations between CD27^-^CD28^-^ CD4^+^ T cell functional parameters and clinical characteristics by linear regression after adjustment for age and sex.

|  | CD107a^+^ | IFN-γ | TNF-α | TNF-α⁺IFN-γ⁺CD107a⁺ |
| --- | --- | --- | --- | --- |
|  | *β (95% CI)* | *β (95% CI)* | *β (95% CI)* | *β (95% CI)* |
| Any Biologic Use | -0.02 (-0.14, 0.11) | -0.03 (-0.14, 0.09) | -0.01 (-0.12, 0.10) | -0.01 (-0.11, 0.09) |
| JAKi Use | -0.07 (-0.21, 0.07) | -0.07 (-0.20, 0.07) | -0.05 (-0.17, 0.07) | -0.04 (-0.15, 0.07) |
| Prednisone Use | -0.08 (-0.31, 0.16) | -0.08 (-0.29, 0.13) | -0.06 (-0.25, 0.13) | -0.06 (-0.23, 0.11) |
| Methotrexate Use | -0.08 (-0.22, 0.06) | -0.08 (-0.21, 0.05) | -0.04 (-0.16, 0.08) | -0.04 (-0.15, 0.07) |
| ACPA Positive | 0.04 (-0.08, 0.17) | 0.07 (-0.05, 0.18) | 0.04 (-0.06, 0.15) | 0.04 (-0.05, 0.13) |
| Erosive Disease | -0.10 (-0.26, 0.06) | -0.08 (-0.23, -0.06) | -0.08 (-0.20, 0.05) | -0.05 (-0.18, 0.07) |
| Smoking | -0.02 (-0.15, 0.12) | -0.05 (-0.18, 0.07) | -0.02 (-0.13, 0.26) | -0.03 (-0.11, 0.25) |

**Table S5: List of CyTOF antibodies**

| Antigen | Metal | Clone | Vendor | Staining |
| --- | --- | --- | --- | --- |
| CD57 | 115 | HCD57 | biolegend | surface - post-cell-ID |
| CD3 | 141 | UCHT1 | BD | surface - post-cell-ID |
| CD56 | 142 | HCD56 | Biolegend | surface - post-cell-ID |
| CD8 | 143 | SK1 | Biolegend | surface - post-cell-ID |
| CD4 | 144 | SK3 | Biolegend | surface - post-cell-ID |
| CD16 | 145 | B73.1 | Biolegend | surface - post-cell-ID |
| Granzyme B | 146 | CLB-GB11 | eBioscience | intracellular |
| CD19 | 147 | HIB19 | Biolegend | surface - post-cell-ID |
| HLA-DR | 148 | L243 | Biolegend | surface - post-cell-ID |
| CD127 | 150 | A019D5 | 351302 | surface - pre cell-ID |
| CD38 | 151 | HIT2 | Biolegend | surface - post-cell-ID |
| CTLA4 | 152 | BNI3 | BD | intracellular |
| CCR2 | 153 | K036C2 | Biolegend | surface - pre cell-ID |
| CD27 | 154 | LG.7F9 | eBioscience | surface - pre cell-ID |
| TCRab | 155 | T10B9.1A-31 | BD | surface - pre cell-ID |
| CXCR3 | 156 | G025H7 | Biolegend | surface - pre cell-ID |
| PD-1 | 158 | EH12.2H7 | Biolegend | surface - post-cell-ID |
| CD28 | 160 | CD28.2 | Biolegend | surface - post-cell-ID |
| CD11b | 162 | ICRF44 | Biolegend | surface - post-cell-ID |
| ICOS | 163 | C398.4A | Biolegend | surface - post-cell-ID |
| Ki67 | 164 | B56 | BD | intracellular |
| Foxp3 | 165 | PCH101 | eBiosciences | intracellular |
| TCF1 | 166 | C63D9 | Cell Signaling Technology | intracellular |
| CD45RA | 167 | HI100 | eBio | surface - post-cell-ID |
| SIRPa | 168 | 15-414 | Biolegend | surface - post-cell-ID |
| Granulysin | 169 | DH2 | Biolegend | intracellular |
| CXCR5 | 170 | RF8B2 | BD | surface - post-cell-ID |
| CD94 | 171 | DX22 | Biolegend | surface - post-cell-ID |
| 2B4 | 172 | C1.7 | Biolegend | surface - post-cell-ID |
| CCR6 | 173 | G034E3 | G034E3 | surface - pre cell-ID |
| CD39 | 174 | ebioA1 | eBiosciences | surface - post-cell-ID |
| perforin | 175 | dG9 | Biolegend | intracellular |
| CD95 | 176 | DX2 | Biolegend | surface - post-cell-ID |

**Table S6: List of flow cytometry antibodies**

| Antigen | Fluorochrome | Clone | Vendor | Catalog # |
| --- | --- | --- | --- | --- |
| CD107a | PE- Dazzle 594 | H4A3 | Biolegend | 328646 |
| Live/Dead | Near-IR | NA | Invitrogen | L10119 |
| CD27 | AF700 | O323 | Biolegend | 302814 |
| CD28 | APC | CD28.2 | Biolegend | 302911 |
| CD45RO | BV650 | UCHL1 | Biolegend | 304231 |
| CD45RO | FITC | UCHL1 | Biolegend | 304242 |
| CD95 | PE-Dazzle 594 | DX2 | Biolegend | 305634 |
| CD3 | BV605 | OKT3 | Biolegend | 317322 |
| CD3 | PE-Cy5 | UCHT1 | Biolegend | 300410 |
| CD4 | BV510 | SK3 | Biolegend | 344634 |
| CD4 | BV650 | OKT4 | Biolegend | 317436 |
| CD8 | PE-Cy5 | HIT8a | Biolegend | 300910 |
| Granzyme B | PE-Cy7 | QA18A28 | Biolegend | 396410 |
| Perforin | PE | B-D48 | Biolegend | 353304 |
| TNF-α | BV785 | MAb11 | Biolegend | 502948 |
| IFN-γ | FITC | 4S.B3 | Biolegend | 502506 |
